# Identification and design principles of far-red–absorbing chlorophyll in the light-harvesting complex

**DOI:** 10.1016/j.jbc.2025.108518

**Published:** 2025-04-18

**Authors:** Keisuke Saito, Makiko Kosugi, Linhao Qiu, Jun Minagawa, Hiroshi Ishikita

**Affiliations:** 1 Department of Applied Chemistry, The University of Tokyo, Tokyo, Japan; 2 Research Center for Advanced Science and Technology, The University of Tokyo, Tokyo, Japan; 3 Division of Environmental Photobiology, National Institute for Basic Biology, Okazaki, Aichi, Japan; 4 Graduate Institute for Advanced Studies, SOKENDAI, Okazaki, Aichi, Japan

**Keywords:** chlorophylls, excitation energy transfer, excitonic coupling, light-harvesting antenna complex, photosynthesis

## Abstract

Photosystem II from *Prasiola crispa* employs a unique ring-shaped undecameric light-harvesting complex (Pc-frLHC) in addition to the commonly observed minor monomeric and major trimeric LHCIIs. Each monomer of Pc-frLHC contains four transmembrane helices. In contrast to the typical three-helix LHCIIs that constitute for the peripheral light-harvesting antennas for photosystem II, Pc-frLHC carries chlorophylls capable of far-red absorption. Combining spectroscopic analyses with a quantum mechanical/molecular mechanical approach, we identified the far-red–absorbing chlorophyll(s) in Pc-frLHC, as well as its counterpart in another Trebouxiophyceae alga *Coccomyxa* sp. Obi (Co-frLHC). Spectroscopic analysis reveals that both complexes exhibit far-red–shifted absorption of chlorophylls at ∼710 nm. In the Pc-frLHC structure, the Chl*a* 603-609 dimer exhibits the strongest excitonic coupling among all apparent chlorophyll dimers. This dimer also exhibits the largest excitation-induced permanent dipole moment along the axis connecting the two chlorophylls, reflecting the most pronounced charge-transfer character. Furthermore, Chl*a* 609 forms the second strongest excitonically coupled dimer with Chl*a* 708, further extending the absorption into the far-red region. The conserved spatial arrangement and orientation of the chlorophyll trimer in Co-frLHC suggest that the Chl*a* 603-609-708 trimer, located in the same frLHC monomer unit, which is predominantly characterized by the Chl*a* 603-609 dimer, provides the structural basis for the far-red absorption in frLHCs.

On Earth, the photon flux density of sunlight is relatively low, on the order of ∼100 mW/cm^2^, and under diffuse light conditions, photons arrive at individual pigment molecules only every few hundred milliseconds (1). To compensate for this limitation and maintain a steady rate of charge separation, photosynthetic organisms employ large numbers of antenna pigments in light-harvesting complexes (LHCs), which increase the effective cross-section for photon capture. These LHCs transfer the captured excitation energy to reaction centers, such as photosystem I (PSI) and photosystem II (PSII), where charge separation occurs and light energy is converted into chemical energy. Thus, the architecture and pigment composition of LHCs critically influence the overall efficiency of photosynthetic energy conversion.

In PSI, a pair of monomeric chlorophylls, P_A_ and P_B_, forms the site with the lowest excitation energy, serving as the initial electron donor, P700, absorbing at 700 nm. This is due to the strong electronic coupling between P_A_ and P_B_ (∼90 meV (2)). While PSI has two electron-transfer–active branches, the A and B branches (3, 4, 5), electron transfer primarily occurs along the A branch (6, 7). In PSII, however, the corresponding chlorophyll pair, P_D1_ and P_D2_, exhibits much weaker electronic coupling (∼10 meV), making it unlikely to serve as the initial electron donor (8). Instead, the monomeric accessory chlorophyll, Chl_D1_, in the active D1 branch serves as the initial electron donor, P680, absorbing at 680 nm (8).

Although charge separation can take place solely in these reaction centers, direct photon absorption by the chlorophylls bound to the reaction centers is significantly limited, even under full sunlight conditions. This limitation arises from the limited number of chlorophylls, typically up to six, present in a reaction center. To overcome this limitation, photosynthetic organisms employ LHCs as peripheral antennas, thereby increasing the number of chlorophylls capable of absorbing light energy. In PSI, light harvesting is achieved through LHCI antennas, which have chlorophylls with red-shifted absorption (red-shifted chlorophylls) (9, 10, 11). Most LHCI complexes are composed of Lhca subunits with three transmembrane helices (three-helix LHC), although some, such as Lhca2 from *Chlamydomonas reinhardtii* (CrLhca2), are composed of four transmembrane helices (four-helix LHC) (12). In PSII, the corresponding peripheral antennas are LHCII complexes, composed of Lhcb/LhcbM subunits with three transmembrane helices, such as Lhcb1-3 in spinach (13) (Fig. 1). Unlike LHCI, LHCII generally lacks red-shifted chlorophylls, consistent with the shorter absorption wavelength of the initial electron donor P680 in PSII than P700 in PSI.

**Figure 1:**
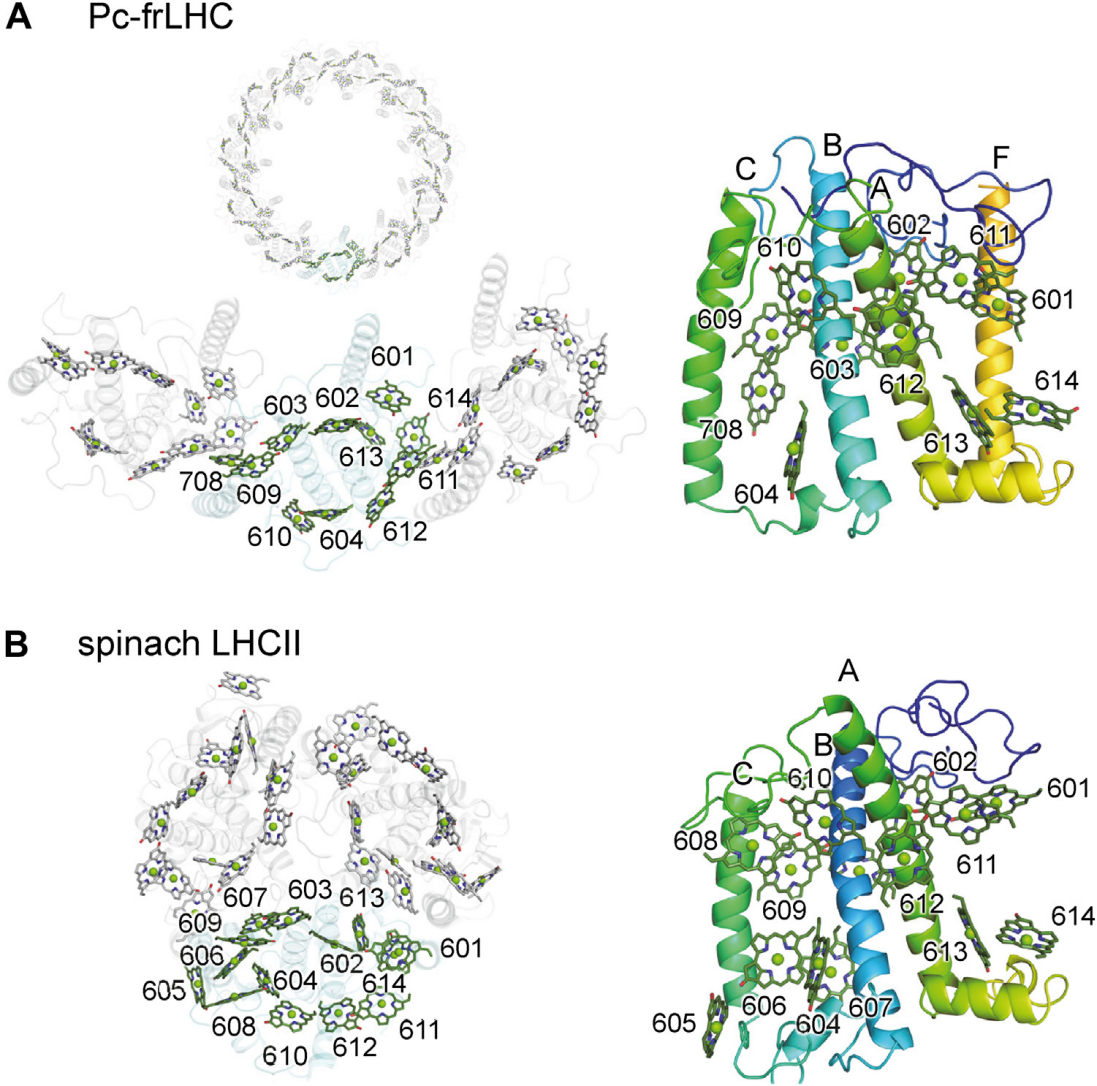
**Overview of three-helix and four-helix LHC structures.***A*, structure of Pc-frLHC complex (PDB: 8HW1). The *upper left panel* shows the overall undecameric complex composed of 11 four-helix frLHC monomers (membrane perpendicular view). The *bottom left panel* highlights one frLHC monomer with labeled chlorophylls. The *right panel* shows a membrane parallel view of a single frLHC monomer with labeled transmembrane helices and chlorophylls. *Labels B*, *C*, *A*, *and F* indicate the first, second, third, and fourth transmembrane helices, respectively. *B*, structure of spinach LHCII (PDB: 1RWT). The *left panel* shows the trimeric arrangement of LHCII composed of three-helix Lhcb monomers, highlighting one LHCII monomer with labeled chlorophylls (membrane perpendicular view). The *right panel* shows an LHCII monomer with labeled transmembrane helices and chlorophylls (membrane parallel view). *Labels B*, *C*, *and A* indicate the first, second, and third transmembrane helices, respectively. LHC, light-harvesting complex.

PSII from *Prasiola crispa*, a dominant green alga of the Trebouxiophyceae class that inhabits Antarctica, employs typical LHCII as peripheral light-harvesting antennas. These include minor monomeric LHCIIs (CP29 and CP26) and major trimeric LHCIIs (Lhcb1-8) (14). In addition, it uses a ring-like undecameric complex (Pc-frLHC) for harvesting far-red light (15). The Pc-frLHC complex is composed of 11 frLHC subunits, each containing four transmembrane helices. The recent genomic information of *P. crispa* strain 4113 revealed that Pc-frLHC is encoded by *frLHC1-4* genes (14). Notably, frLHC1-4 are orthologous to a noncanonical member of LHCI with four transmembrane helices, such as Lhca2 in *C. reinhardtii* (16). In contrast to typical LHCII, the Pc-frLHC complex exhibits significantly red-shifted chlorophyll absorption, peaking around 710 nm (17). Although the far-red–shifted chlorophylls in the Pc-frLHC complex might appear disadvantageous for delivering excitation energy to P680 in PSII, they may confer an adaptive advantage as several Trebouxiophyceae algae also possess *frLHC* genes (14). *P. crispa* is commonly observed to form layered colonies, with cells in the deeper layers exposed to limited visible light. Even in these deeper cell layers, far-red light remains reachable. Thus, the ability to absorb far-red light is likely essential for sustaining photosynthesis in *P. crispa* (18, 19, 20).

The cryo-electron microscopy (EM) structure of the Pc-frLHC complex potentially provide insights into the mechanisms enabling far-red light absorption and its energy transfer to PSII *via* an energetically uphill process (15). The Pc-frLHC structure exhibits 11-fold symmetry, consisting of 11 of frLHC monomers (Fig. 1). Identifying the far-red–shifted chlorophyll(s) (∼710 nm absorption) is essential for understanding the structural framework for far-red light absorption and the mechanism by which its energy is transferred to PSII. Based on the resolved structure of the Pc-frLHC complex, five chlorophylls located at the interface between frLHC monomers (Chl*a* 603, Chl*a* 609, and Chl*a* 708 in one frLHC and Chl*a* 613 and Chl*a* 614 in the adjacent frLHC) were proposed to form an excitonically coupled pentamer responsible for far-red absorption (15). While excitonically coupled chlorophyll dimers are known to exhibit redshift absorption (21), it remains unclear whether the proposed chlorophylls bound to frLHC can form a pentameric structure capable of producing the observed far-red absorption. Comparative analysis with orthologous frLHCs, such as frLHC from *Coccomyxa* sp. Obi (Co-frLHC), another green alga in the Trebouxiophyceae class (15), could provide further insights into the mechanisms underlying red-shifted chlorophyll absorption.

Here, we report the origin of the far-red chlorophyll in the Pc-frLHC structure by combining spectroscopic analyses of Pc-frLHC and Co-frLHC with a quantum mechanical/molecular mechanical (QM/MM) approach.

## Results and discussion

### Absorption wavelengths of frLHC complexes

**Figure 2:**
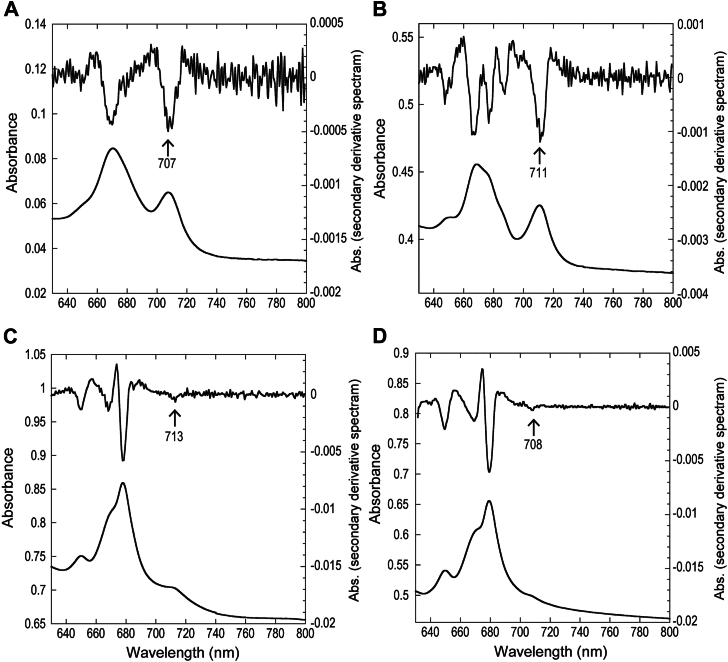
**Absorbance spectra and secondary derivative spectra around the Qy band.***A*, purified Pc-frLHC at 298 K. *B*, purified Pc-frLHC at 93 K. *C*, isolated thylakoid membranes from *P. crispa* at 93 K. *D*, isolated thylakoid membranes from *Coccomyxa* sp. Obi at 93 K. The arrows and numbers indicate the peak wavelengths (in nm) of long-wavelength chlorophylls. See Figure S1 for the original absorbance spectra covering 370 to 800 nm. The spectra shown are representative of multiple independent measurements that gave consistent results. LHC, light-harvesting complex.

The spectral properties of the red-shifted chlorophylls in the Pc-frLHC and Co-frLHC complexes were investigated by measuring their absorption (Figs. 2 and S1) and fluorescence emission spectra (Fig. S2). In the purified Pc-frLHC complex, an absorption peak was identified at 707 nm at 298 K (Fig. 2*A*), which is consistent with previous reports (15). At cryogenic temperatures, this peak red-shifted slightly to 711 nm (Fig. 2*B*). The far-red absorption peak of the *P. crispa* thylakoids was found at 713 nm (Fig. 2*C*). The minor redshift observed relative to the purified sample is likely due to the lower proportion of far-red–absorbing chlorophylls bound to Pc-frLHC among those associated with other complexes, such as LHCI and LHCII. Similarly, thylakoids from *Coccomyxa* exhibited a far-red absorption peak, which was observed at a slightly blue-shifted position compared to the *P. crispa* thylakoids.

The fluorescence emission peaks of the isolated frLHC complex as well as the thylakoids from *P. crispa* were observed at 713 nm at 298 K and 730 nm at 77 K (Fig. S2), as previously reported (15, 17). Notably, under cryogenic conditions, the far-red fluorescence emission from the thylakoid membranes originates not only from frLHC but also from LHCI(s). Therefore, the observed far-red fluorescence in the thylakoid membranes likely results from overlapping contributions of both frLHC and LHCI, as indicated by the distinct emission peaks. The far-red fluorescence emission peak in *Coccomyxa* thylakoid membranes at 298 K, which was absent in the cells under white light, can be attributed to the Co-frLHC complex, as frLHC is not expressed under white light (Table 1). These red-shifted chlorophylls were not detected in CrLhca2 (Table 1) despite it belongs to the same four-helix LHC clade as frLHC1-4. This indicates that the frLHC1-4 in *P. crispa* and in *Coccomyxa* sp. Obi acquired red-shifted chlorophylls following the divergence of Trebouxiophyceae and Chlorophyceae in the evolutionary history of green algae.

**Table 1:** Absorption and fluorescence emission bands of frLHC complex and thylakoids in the far-red region

	Temperature (K)	Absorption (nm)	Fluorescence (nm)
Pc-frLHC (purified)	298	707	713
	93	711	–
	77	–	733
*P. crispa* thylakoids	298	–	713
	93	713	–
	77	–	733
*Coccomyxa* thylakoids	298	–	710
	93	708	–
	77	–	709-716
CrLhca2^a^	77	693-697	717

The peaks were identified at the indicated measuring temperatures through the second derivative of the spectrum. –, not detected.

a Ref. (11).

It should be noted that absorption spectra were measured at 93 K to ensure thermal stability and prevent condensation in the temperature-controlled setup. While fluorescence spectra were recorded at 77 K, a comparison with absorption data collected at 123 K revealed that the red-shifted peak positions at 93 K differ by less than 1 nm, confirming their robustness. At 93 K, thermal energy is insufficient to induce large-scale structural fluctuations, and vibrational excitation is largely frozen out. Since quantum chemical calculations, including QM/MM calculations, inherently describe the electronic structure at 0 K and the optimized geometry reflects the dominant conformational state at cryogenic temperature, the 93 K spectra are qualitatively appropriate for comparison.

### Absorption wavelengths of the monomer chlorophylls in Pc-frLHC

To identify the chlorophyll(s) responsible for the experimentally measured longest absorption wavelength in the Pc-frLHC complex, absorption wavelengths of each chlorophyll bound to the Pc-frLHC polypeptide were calculated using a QM/MM/polarizable continuum model (PCM) approach. The calculated absorption wavelengths for the 11 distinct monomer chlorophylls range from 664 nm to 690 nm (Tables 2, S1).

**Table 2:** Calculated absorption wavelengths (*λ*_abs_) for monomeric, dimeric, and trimeric chlorophylls in the Pc-frLHC structure (nm)

Chlorophyll	Ligand	*λ*_abs_				Δ*λ*_abs_		
			(Water)	Shift	-Charge	-Charge	-Solvation	-Ring
				(Total)	(Ligand)	(Others)	Loss	Distortion
601	–	681	(681)	0	0	4	−11	7
602	Glu124	664	(681)	−17	5	2	−16	−8
603	Asn127	666	(681)	−15	−2	−1	−20	8
604	–	669	(681)	−12	0	2	−9	−4
609	Glu178	686	(681)	5	10	−6	−13	14
610	Glu219	688	(681)	7	10	−1	−16	13
611	Glu63	671	(681)	−10	4	−2	−20	8
612	Asn222	679	(681)	−2	0	3	−16	10
613	Gln236	664	(681)	−17	0	3	−19	0
614	His251	690	(681)	9	4	−3	−5	13
708	His171	668	(681)	−13	0	−4	−12	3
603-609	–	706	(681)	25	–	–	–	–
609-708	–	699	(681)	18	–	–	–	–
611-612	–	685	(681)	4	–	–	–	–
613-614	–	692	(681)	11	–	–	–	–
708-614’	–	691	(681)	10	–	–	–	–
603-609-708	–	711	(681)	30	–	–	–	–

The shifts in *λ*_abs_ from water (681 nm) to the Pc-frLHC protein environment (Δ*λ*_abs_) are decomposed into contributions from: 1) atomic charges of the axial ligand group, 2) atomic charges of the other protein environment, 3) loss of solvation at the binding site in the protein environment, and 4) distortion of chlorin ring caused by the protein environment. In the present case, Δ*λ*_abs_ is practically the sum of these four components. –, not determined. See Tables S1 and S6 for details.

Among the factors influencing chlorophyll absorption wavelengths in the Pc-frLHC protein environment relative to bulk water, the “loss of solvation” effect consistently induces a blueshift in all chlorophylls (up to 20 nm, with an average of 14 nm; Table 2). This suggests that, unless counterbalanced by other factors, chlorophylls are inherently blue-shifted in the protein environment. In contrast, these blueshifts are partially offset by redshifts caused by electrostatic interactions with axial ligand groups (see Table S2 for a list of these ligands), particularly with glutamate (up to 10 nm; Table 2). If the axial ligand group is glutamate, the surrounding “other protein environment,” which tends to position basic residues near the negatively charged ligand (*e.g.*, Arg181 near Glu178, the axial ligand of Chl*a* 609, see below), in turn, contribute to blueshifts. However, the influence of the “other protein environment” is much smaller than that of the axial ligand group (with an average of 0 nm; Table 2), as the other protein components are located farther from the chlorophyll compared to its axial ligand group. The most significant contributor to redshifts, however, is “chlorin ring distortion” induced by the protein environment (up to 14 nm, with an average of 6 nm; Table 2). Notably, the majority of chlorophylls (nine out of eleven) are red-shifted due to this distortion.

In the Pc-frLHC protein structure, only three chlorophylls, Chl*a* 609 (5 nm redshift), Chl*a* 610 (7 nm redshift), and Chl*a* 614 (9 nm redshift), exhibit redshifts. However, their absorption wavelengths (<690 nm; Table 2) remain insufficient to account for the experimentally observed absorption wavelength of ∼710 nm in the Pc-frLHC complex (Table 1). These results suggest that while “chlorin ring distortion” and specific “axial ligand” interactions contribute to redshifts in chlorophylls in the protein environment, their effects are largely offset by the blueshifting due to “loss of solvation.” Thus, additional molecular mechanisms, beyond those considered here, must be responsible for the observed long-wavelength absorption in the Pc-frLHC complex.

### Absorption wavelengths of multimer chlorophylls in the Pc-frLHC complex

In addition to the factors discussed above, if two chlorophylls are in close proximity, their excitonic coupling becomes significant, resulting in the formation of an excitonically coupled, red-shifted chlorophyll dimer (*e.g.*, the special pair bacteriochlorophylls in reaction centers from purple bacteria (8)). The Pc-frLHC structure reveals five potential chlorophyll dimers, where the edge-to-edge distance between the two chlorophylls is <∼5 Å. All five chlorophyll dimers investigated exhibit redshifts compared to their monomeric chlorophyll forms (Table 2).

**Figure 3:**
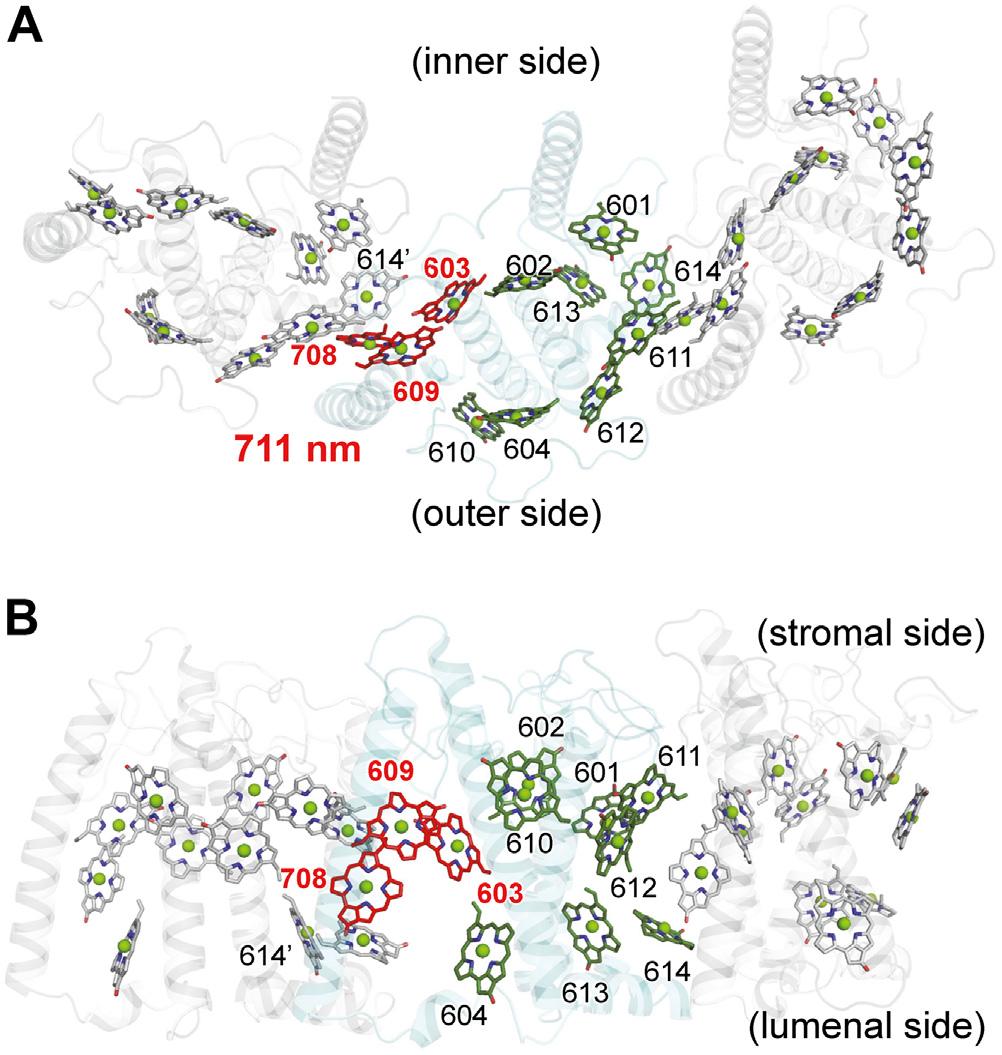
**Location of red-shifted chlorophylls in the Pc-frLHC.***A*, view along the membrane vertical axis. *B*, *horizontal view* along the membrane plane. Chl*a* 613′ and Chl*a* 614′ denote Chl*a* 613 and Chl*a* 614 in an adjacent Pc-frLHC monomer unit, respectively. Chlorophylls in the Pc-frLHC monomer unit in focus are highlighted in *red* (Chl*a* 603-609-708 trimer) or *green* (other chlorophylls). Chlorophylls in adjacent LHC monomer units are shown in *gray*. LHC, light-harvesting complex.

Remarkably, the Chl*a* 603-609 dimer shows the largest redshift, resulting in an absorption wavelength at 706 nm (Table 2). While this redshift in the Chl*a* 603-609 dimer may already be sufficient to account for the experimentally measured absorption wavelength of ∼710 nm in the Pc-frLHC complex (Table 1), it is interesting to note that the chlorophyll dimer exhibiting the second-largest redshift, the Chl*a* 609-708 dimer (699 nm), also involves Chl*a* 609. Given that Chl*a* 609 is shared with both Chl*a* 603 and Chl*a* 708, it seems worthwhile to investigate whether these three chlorophylls, Chl*a* 603, Chl*a* 609, and Chl*a* 708, form an excitonically coupled chlorophyll trimer. QM/MM/PCM calculations indicate that the absorption wavelength of the Chl*a* 603-609-708 trimer is further red-shifted to 711 nm (Table 2 and Fig. 3), aligning closely with the experimentally measured absorption wavelength of ∼710 nm in the Pc-frLHC complex (Table 1).

In previous studies, a chlorophyll pentamer (Chl*a* [603-609-708]-[613′-614’]), incorporating two additional monomeric chlorophylls (Chl*a* 613 and Chl*a* 614 from an adjacent LHC monomer unit, denoted as Chl*a* 613′ and Chl*a* 614′, respectively) alongside the Chl*a* 603-609-708 trimer, was hypothesized as a potential contributor to the far-red–shifted chlorophyll in the Pc-frLHC (15). However, while the formation of the Chl*a* 603-609 dimer (706 nm; +20 nm redshift from 686 nm for Chl*a* 609) and the Chl*a* 609-708 dimer (699 nm; +13 nm redshift from 688 nm for Chl*a* 708) results in significant redshifts, the formation of the Chl*a* 613′-614′ dimer (692 nm; +2 nm redshift from 690 nm for Chl*a* 614) and the Chl*a* 708-614′ dimer (691 nm; +1 nm redshift from 690 nm for Chl*a* 614) results in only marginal redshifts (Table 1). These findings indicate that the far-red–shifted chlorophyll can be substantially explained by the Chl*a* 603-609-708 trimer alone, without requiring an extension to a chlorophyll pentamer.

Overall, in addition to tuning the “protein environment” through factors such as “chlorin ring distortion” or specific “axial ligand” interactions, tuning the chromophore itself *via* dimerization or trimerization is a likely strategy used by the protein to achieve far-red–shifted chlorophylls.

### Mechanism of far-red–shifted absorption in the Chla 603-609-708 trimer

**Figure 4:**
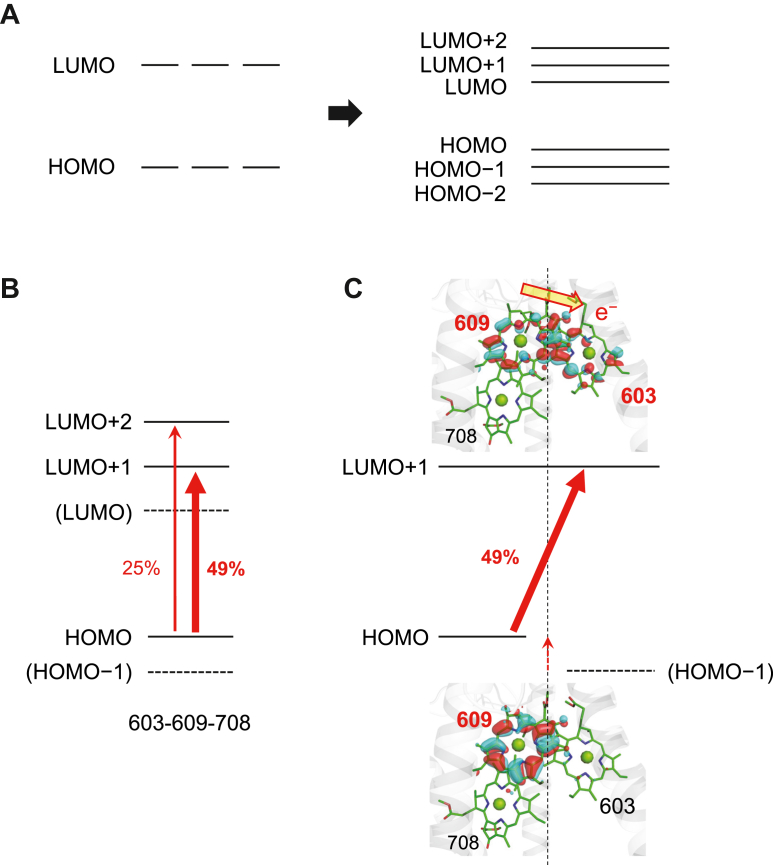
**Molecular orbitals of the Chl*a* 603-609-708 trimer.***A*, [HOMO] and [LUMO] of the three representative monomeric chlorophylls, which hybridize to form [HOMO−2], [HOMO−1], [HOMO], [LUMO], [LUMO+1], and [LUMO+2] in the chlorophyll trimer. *B*, transitions contributing to the lowest excited state of the Chl*a* 603-609-708 trimer, specifically [HOMO] to [LUMO+1] (49%) and [HOMO] to [LUMO+2] (25%). *C*, energy levels and spatial distribution of [HOMO] and [LUMO+1], the major orbitals responsible for the absorption wavelength of the Chl*a* 603-609-708 trimer. *Red thick arrows* indicate transitions upon electronic excitation. The *red dotted vertical arrow* indicates the destabilization of [HOMO] localized in the Chl*a* 609 region due to its negatively charged axial ligand, Glu178, resulting in the [HOMO] of the Chl*a* 603-609-708 trimer. In contrast, the destabilization is absent for Chl*a* 603 due to its charge-neutral axial ligand, Asn127, resulting in a more stable [HOMO−1] localized in the Chl*a* 603 region of the trimer. HOMO, highest occupied molecular orbital; LUMO, lowest unoccupied molecular orbital.

In a monomeric chlorophyll, the Qy absorption band is primarily associated with the highest occupied molecular orbital [HOMO] to lowest unoccupied molecular orbital [LUMO] electronic transition (22). However, in the chlorophyll trimer, the Qy absorption band involves the hybridization of molecular orbitals from the three monomeric chlorophylls, forming three HOMOs [HOMO], [HOMO−1], and [HOMO−2] and three LUMOs [LUMO], [LUMO+1], [LUMO+2] (Fig. 4*A*). According to QM/MM/PCM calculation, the lowest excited state of the Chl*a* 603-609-708 trimer is predominantly composed of the [HOMO] to [LUMO+1] (49%) and [HOMO] to [LUMO+2] (25%) transitions (Fig. 4*B*).

Importantly, the [HOMO] in the trimer is localized at Chl*a* 609, while the [LUMO+1] and [LUMO+2] are delocalized over both Chl*a* 603 and Chl*a* 609. Thus, upon electronic excitation, electron transfer occurs from the [HOMO] of the Chl*a* 603-609 dimer, which is localized on Chl*a* 609, to the [LUMO+1] and [LUMO+2], which are delocalized over the Chl*a* 603-609 dimer. This process gives rise to a “charge-transfer” character within the trimer (Fig. 4*C*). In this context, the charge-transfer nature of the Chl*a* 603-609-708 trimer is predominantly inherited from the Chl*a* 603-609 dimer.

**Figure 5:**
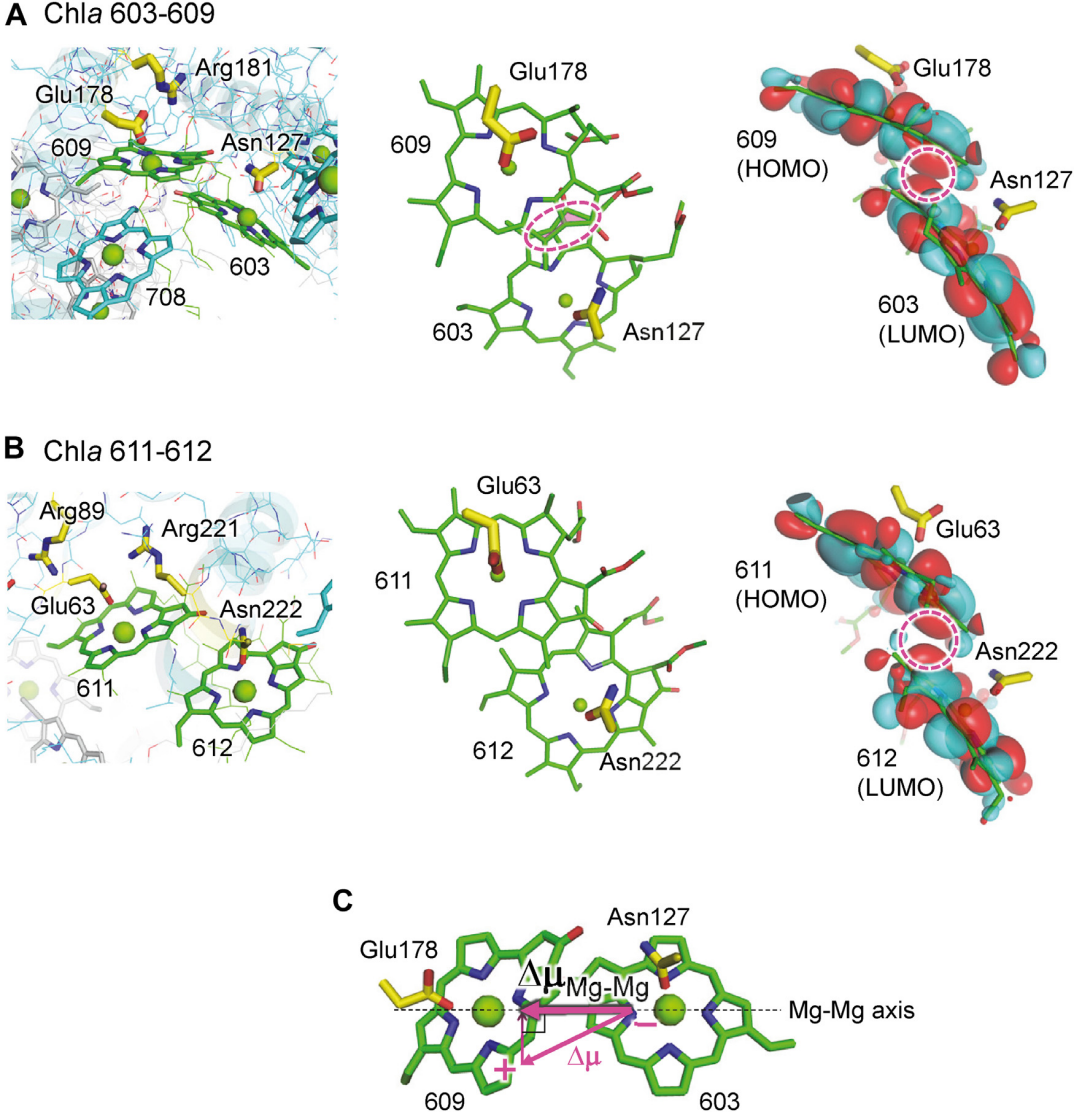
**Chlorophyll dimers in the Pc-frLHC structure.***A*, Chl*a* 603-609 dimer. *B*, Chl*a* 611-612 dimer. *Left panels*: protein environment near the chlorophyll dimer binding sites. *Middle panels*: overlap of C=C bonds between two monomeric chlorophylls. *Right panels*: spatial distribution of the [HOMO] of the glutamine-ligated chlorophyll (Chl*a* 609 and Chl*a* 611) and the [LUMO] of the asparagine-ligated chlorophyll (Chl*a* 603 and Chl*a* 612), shown simultaneously. Importantly, overlap between the [HOMO] of the donor chlorophyll (*e.g.*, Chl*a* 609) and the [LUMO] of the acceptor chlorophyll (*e.g.*, Chl*a* 603) is critical for excitation-induced charge transfer. The more pronounced orbital overlap in the Chl*a* 603-609 dimer compared to the Chl*a* 611-612 dimer (*pink dotted circles*) explains the stronger charge-transfer character in the Chl*a* 603-609 dimer. *C*, excitation-induced permanent dipole moment of the chlorophyll dimer, Δμ, and its projection, Δμ_Mg-Mg_, along the axis connecting the two chlorophylls (*i.e.*, Mg–Mg axis). The magnitude of Δμ_Mg-Mg_ more directly reflects charge transfer and its contribution to *red*-shift absorption than Δμ. HOMO, highest occupied molecular orbital; LHC, light-harvesting complex; LUMO, lowest unoccupied molecular orbital.

The localization of the [HOMO] at Chl*a* 609 is due to the difference in the electrostatic environments of the two monomeric chlorophyll, specifically their axial ligands. Chl*a* 609, coordinated by negatively charged Glu178, has its [HOMO] and [LUMO] destabilized compared to Chl*a* 603, coordinated by charge-neutral Asn127 (Fig. 5). Consequently, the [HOMO] of the dimer reflects the high-energy character of Chl*a* 609, leading to electron excitation originating predominantly from the Chl*a* 609. It should be noted that the delocalization of the [LUMO+1] and [LUMO+2] over Chl*a* 603 and Chl*a* 609 is less influenced by the axial ligands because the electrostatic effects are less crucial for unoccupied molecular orbitals. Therefore, while the [HOMO] is localized at Chl*a* 609, the [LUMO+1] and [LUMO+2] are shared between both Chl*a* 603 and Chl*a* 609.

This electronic excitation involving charge transfer decreases the excitation energy of the Chl*a* 603-609 dimer, resulting in a red-shifted absorption. The utilization of the destabilized [HOMO] of Chl*a* 609, combined with the delocalization of unoccupied orbitals, is a key mechanism for the observed redshift in the Chl*a* 603-609 dimer.

Remarkably, the charge-transfer character observed in the red-shifted Chl*a* 603-609 dimer in Pc-frLHC, where the [LUMO+1] is delocalized over the chlorophyll dimer while the [HOMO] is predominantly localized on one of the two monomeric chlorophylls, significantly contrasts with the red-shifted P700 chlorophyll dimer in the PSI reaction center. In the P700 dimer, composed of the monomeric P_A_ and P_B_ chlorophylls, the [HOMO] is delocalized over the chlorophyll dimer, whereas the [LUMO] is predominantly localized on one of the monomeric chlorophylls, P_A_ (2). Despite these differences, both chlorophyll dimers exhibit charge-transfer character, indicating that both monomeric and dimeric properties must coexist in the ground and excited states to enable charge-transfer–driven red-shifted chlorophyll dimers. If only dimeric properties were present, the charge-transfer character would disappear. Conversely, if only monomeric properties were present, the transition dipole moment would vanish, rendering the excitation optically forbidden.

In Pc-frLHC, destabilization of the [HOMO] of the monomeric chlorophyll is achieved through its negatively charged axial ligand. In contrast, in the PSI reaction center, localization of the [LUMO] on P_A_ is achieved *via* chlorophyll epimerization (*i.e.*, 13^2^-epimer chlorophyll at P_A_ (2, 23)). These distinct approaches for inducing charge transfer appear to arise from differences in protein architecture. In Pc-frLHC in *P. crispa*, the two axial ligand residues for the Chl*a* 603-609 dimer are located on the same protein polypeptide. In the PSI reaction center, however, the two axial ligand residues for the [P_A_P_B_] dimer are located on separate polypeptides, PsaA and PsaB. Given the dimeric nature of the reaction center, the amino acid sequences of PsaA and PsaB exhibit a high similarity, particularly in the fully conserved axial ligand residues for the chlorophylls P, A_−1A_, and A_0_. This structural symmetry makes it more practical for the PSI reaction center to form a charge-transfer red-shifted chlorophyll dimer *via* epimerization of one of the chlorophylls.

The localization of the [LUMO] of P700 on the monomeric chlorophyll P_A_ in the PSI reaction center enhances its overlap with the [LUMO] of the electron acceptor, A_−1A_, facilitating rapid electron transfer to the electron-acceptor accessory chlorophyll, A_−1_, during charge separation. However, in Pc-frLHC, such localization of the [LUMO] on one of the monomeric chlorophylls in the Chl*a* 603-609 dimer (or [LUMO+1] in the Chl*a* 603-609-708 trimer]) could potentially lead to charge separation and disrupt excitation energy transfer toward PSII.

These distinct mechanisms for redshift absorption *via* charge transfer likely reflect the unique functional roles of these chlorophyll dimers in the antenna complex and the reaction center.

### Key factors driving far-red absorption *via* charge transfer in the Pc-frLHC structure

Two major factors contribute to the charge-transfer character and, consequently, the far-red absorption of the Chl*a* 603-609-708 trimer.

#### Strong excitonic coupling

The Chl*a* 603-609 dimer exhibits the strongest excitonic coupling among all chlorophyll dimers in the Pc-frLHC structure, as evidenced by its large redshift relative to typical chlorophyll *a* (25 nm), as well as the significant redshifts of the individual monomers upon dimerization (40 nm for Chl*a* 603 and 20 nm for Chl*a* 609; Table 2). This strong coupling is a critical prerequisite for charge transfer. Importantly, charge transfer between two chlorophylls requires the C=C bonds of the chlorophylls to be close enough to allow overlap between their π-orbitals. Indeed, the *Prasiola* LHC structure exhibits sufficient overlap of the C=C bonds between Chl*a* 603 and Chl*a* 609 compared to other Chl*a* dimers (Fig. 5, *A* and *B*).

#### Electrostatically heterogeneous environments as a driving force for charge transfer

The driving force for charge transfer arises from the electrostatically heterogeneous ligand environments of Chl*a* 603 and Chl*a* 609. Chl*a* 609, coordinated by negatively charged Glu178, has a higher [HOMO] energy level than Chl*a* 603, coordinated by charge-neutral Asn127. This electrostatic asymmetry establishes the energetic basis for charge transfer, enabling the transition from the higher energy [HOMO] on Chl*a* 609 to the [LUMO+1] and [LUMO+2], thereby reducing the excitation energy and resulting in a redshift (Fig. 4).

The involvement of charge transfer upon electronic excitation, which results in a rearrangement of atomic charges over chlorophyll ring planes, can be assessed by examining the excitation-induced permanent dipole moment of the chlorophyll dimer, Δμ. In particular, the dipole moment projected along the axis connecting the two chlorophylls (*i.e.*, Mg–Mg axis), Δμ_Mg-Mg_ provides a more direct representation of charge transfer and its contribution to redshift absorption (Fig. 5*C*). Evaluating charge transfer in chlorophyll dimers using Δμ_Mg-Mg_ is a more relevant approach than relying on other quantities, such as changes in the net charge of each monomeric chlorophyll (see below).

Indeed, the Chl*a* 603-609 dimer exhibits the largest Δμ_Mg-Mg_ (∼3 Debye) among all chlorophyll dimers in the Pc-frLHC structure (Table S3), reflecting its strong charge-transfer character. For comparison, the Chl*a* 613-614 dimer, exhibiting a smaller redshift (Table 2), has a smaller Δμ_Mg-Mg_ (∼1 Debye). This weaker charge-transfer character is due to the electrostatically similar ligand environments of its monomers (charge-neutral glutamine and histidine; Table S3).

The relevance of evaluating charge-transfer character using Δμ_Mg-Mg_ is further demonstrated by the Chl*a* 611-612 dimer. Although this dimer has heterogeneous ligand environments (Glu63 and Asn222), similar to the Chl*a* 603-609 dimer, its Δμ_Mg-Mg_ is smaller (∼1 Debye; Table S3), indicating weaker charge-transfer character. This weaker charge-transfer character is primarily due to a smaller overlap of molecular orbitals than the Chl*a* 603-609 dimer. In addition, the presence of two positively charged arginine residues near Glu63 compensates for the heterogeneous ligand environments, further diminishing the charge-transfer character (Fig. 5*B*).

While our QM/MM/PCM calculations suggest that charge-transfer character in the Chl*a* 603-609 dimer plays a key role in red-shifted absorption, direct experimental confirmation remains an important challenge. Stark spectroscopy, which can detect excitation-induced dipole moment changes (*e.g.*, as demonstrated in the LH1 and LH2 antenna complexes (24)), could in principle validate the charge-transfer character suggested from the calculated Δμ_Mg-Mg_ value. Time-resolved fluorescence measurements may also provide complementary insights by identifying charge-transfer states (25, 26).

### Implications from other LHCs

The present study identifies the Chl*a* 603-609-708 trimer, particularly the Chl*a* 603-609 dimer, as the most plausible origin of far-red absorption in Pc-frLHC. Notably, in Lhca3 and Lhca4 from *Arabidopsis thaliana* (AtLhca3 and AtLhca4) (27), which have red chlorophylls (10, 28, 29) but lack Chl*a* 708, the Chl*a* 603-609 dimer is present and, based on its structural location, it has been proposed as the origin of far-red–shifted chlorophylls^3^. The Chl*a* 603-609 dimer in AtLhca3 and AtLhca4 also exhibited charge-transfer character upon electronic excitation (30, 31), consistent with the observations in the Pc-frLHC (Fig. 4*C*). However, Pc-frLHC is composed of four transmembrane helices, whereas AtLhca3 and AtLhca4 are composed of only three transmembrane helices, lacking the fourth helix present in the Pc-frLHC structure. Although the fourth helix does not provide axial ligands for chlorophylls or carotenoid binding sites, this structural difference may limit direct comparisons in understanding the origin of far-red chlorophylls in frLHC.

**Figure 6:**
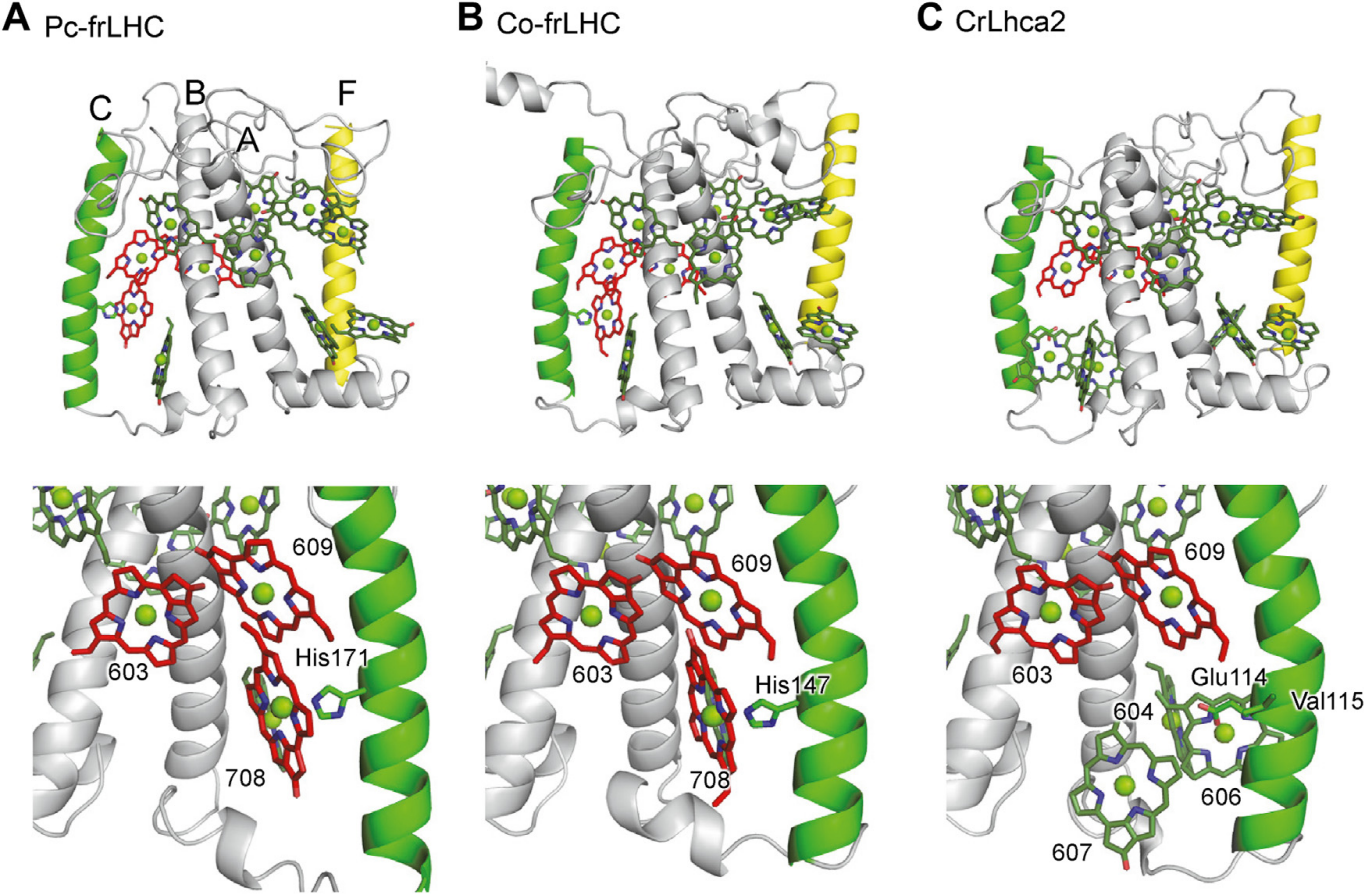
**Four-helix LHC structures.***A*, Cryo-EM structure of Pc-frLHC (PDB: 8HW1). *B*, predicted structure of Co-frLHC (BDA45444.1) using AlphaFold3 (32). *C*, Cryo-EM structure of CrLhca2 structure (PDB: 7DZ7). The Chl*a* 603-609 dimer and adjacent Chl*a* 708 are highlighted in *red*. *Top panels* provide an overview of the entire LHC monomer-unit structures. *Bottom panels* focus on the Chl*a* 603-609 dimer binding regions. *Labels 1 to 4* represent the first to fourth transmembrane helices. The second helix, which provides the axial ligand to Chl*a* 708, is highlighted in *green*. The fourth helix, absent in three-helix LHCs (*e.g.*, AtLhca3 and AtLhca4) is highlighted in *yellow*. LHC, light-harvesting complex; CrLhca2, Lhca2 from *Chlamydomonas reinhardtii.*

In the context of far-red absorption in frLHC, Co-frLHC provides a more relevant comparison (Table 1). While the structure of Co-frLHC has not yet been reported, structural predictions based on amino acid sequences using AlphaFold3 (32) indicate that the protein structure of frLHC shares the same characteristics between *P. crispa* and *Coccomyxa*, in particular the four transmembrane helices (Fig. 6*A*; SI atomic coordinates). Indeed, the axial ligands for the Chl*a* 603-609 dimer (Asn127 and Glu178 in Pc-frLHC and Asn104 and Glu154 in Co-frLHC) and the axial ligand for Chl*a* 708 (His171 in Pc-frLHC and His141 in Co-frLHC) are conserved. This strongly suggests that the red-shifted Chl*a* 603-609-708 trimer in Pc-frLHC is also conserved in *Coccomyxa* (Fig. 6*B*). Since Co-frLHC is one of the few four-helix LHCs reported to exhibit far-red absorption (Table 1), these findings support the conclusion that the Chl*a* 603-609-708 trimer is the origin of far-red–shifted absorption in frLHC in both *P.crispa* and *Coccomyxa*.

While the Chl*a* 603-609 dimer serve as the core of far-red absorption in both three-helix LHCIs (*e.g.*, AtLhca3 and AtLhca4) and four-helix frLHCs (*e.g.*, *P. crispa* and *Coccomyxa*), the presence of Chl*a* 708 and the formation of the Chl*a* 603-609-708 trimer are prerequisites specifically for four-helix LHCs to achieve far-red absorption. This is evident from CrLhca2 (Fig. 6*C*), which shares the Chl*a* 603-609 dimer and the four-helix structure with frLHC in *P. crispa* and *Coccomyxa* but lacks Chl*a* 708 due to the substitution of the histidine ligand with valine (Val115) (33). The absence of far-red absorption in CrLhca2 (Table 1), despite its structural similarity to frLHC except for Chl*a* 708, indicates the critical role of Chl*a* 708 in enabling further red-shifted absorption beyond that of the chlorophyll dimer.

## Conclusions

The present study reveals that far-red chlorophylls exist not only in the Pc-frLHC but also in the Co-frLHC (Table 1). In Pc-frLHC, the Chl*a* 603-609-708 trimer, inherited from the Chl*a* 603-609 dimer, is identified as the origin of the far-red absorption (Table 2). For monomeric chlorophylls in protein environments, the “loss of solvation” effect induces a blueshift (Table 2). In contrast, the most significant contributors to redshifts is primarily “chlorin ring distortion” induced by the protein environment and secondarily electrostatic interactions with “axial ligands,” particularly with negatively charged glutamate.

While these factors modulate the absorption wavelengths of individual monomeric chlorophylls, dimerization or trimerization of chlorophyll has a much larger impact. This indicates that achieving far-red–shifted chlorophylls in LHCs requires not only tuning the “protein environment” (*e.g.*, through “chlorin ring distortion” or specific “axial ligand” interactions) but also tuning the chromophore itself through dimerization or trimerization. In this context, the formation of a far-red–shifted chlorophyll does not necessarily require the two most red-shifted monomeric chlorophylls (Table 2). Instead, the key factor is the formation of a chlorophyll dimer or trimer with charge-transfer character upon electronic excitation.

To enhance charge-transfer character in a chlorophyll dimer, two critical requirements are identified: 1) strong excitonic coupling (*e.g.*, between Chl*a* 603 and Chl*a* 609) facilitated by the overlap of their π-orbitals (pink dotted circles in Fig. 5*A*) and 2) electrostatically heterogeneous ligand environments (*e.g.*, charge-neutral asparagine for Chl*a* 603 and negatively charged glutamate for Chla 609) (Fig. 4*C*, Table S3). In addition, the inclusion of Chl*a* 708 in the trimer further enhances molecular orbital delocalization, resulting in a far-red absorption wavelength.

The conservation of all these key characteristics between Pc-frLHC and CO-frLHC demonstrates that both the electronic structure of the chromophores and the tuning provided by the protein environment are essential for achieving far-red absorption. These findings provide practical criteria for identifying red-shifted chlorophylls in LHCs.

## Experimental procedures

### Spectroscopic measurement

Pc-frLHC sample was prepared from *P*. *crispa* cells collected in Antarctica as described previously (15). *Coccomyxa* sp. Obi cells were cultivated with Bold’s basal medium (34) under 100 μmol photons m^−2^ s^−1^ fluorescence light at 15 °C. To induce red-shifted chlorophylls in *Coccomyxa* sp. Obi, the cells were incubated for several weeks under far-red light emitting diode illumination with a peak wavelength of 732 nm (Fig. S3). Harvested *Coccomyxa* cells suspended in a 0.33 M SM buffer containing 25 mM Mes-NaOH (pH 6.5), 0.33 M sucrose, 5 mM MgCl_2_, and 1.5 mM NaCl were put into a 50 ml conical tube. The cells were disrupted by vortexing with 0.5 mm zirconia/silica beads for 5 s, repeated 10 times with 2-min intervals. The disrupted cells were centrifuged at 700*g* at 4 °C for 4 min and the supernatant was saved as thylakoid membrane. The precipitation was disrupted by bead beating again and thylakoid membranes were isolated by centrifugation at 700*g* for 4 min at 4 °C. All thylakoid membranes were combined and centrifuged at 20,000*g* for 12 min at 4 °C. The resultant precipitation was collected as the thylakoid sample. The Pc-frLHC sample used in this study was highly purified under the same conditions as the cryo-EM sample reported in ref. (15). Pigment analysis confirmed that only violaxanthin and loroxanthin were present in a 1:1 ratio, and no additional carotenoids or pigment–protein complexes were detected. Thus, contamination from other pigments was minimal and did not influence the spectroscopic measurements of Pc-frLHC.

For spectroscopic analyses, the thylakoid and protein samples were diluted to concentrations below 0.1 mg/ml for absorption measurements and 5 μg chlorophyll/ml for fluorescence emission measurements. These concentrations were chosen to minimize LHC aggregation and to avoid self-absorption effects in fluorescence spectra. Absorbance spectra were measured by a UV-visible Spectrophotometer, V-650 (JASCO) with cryostat (Coolspek USP-203, Unisok Co., Ltd). Thylakoid/protein samples containing 60% glycerol were placed into a custom-made cell constructed with two crystal glass plates (10 × 10 × 1 mm) separated by a 0.2 mm thick U-shaped plastic spacer. The slit width was set at 2.0 nm. Baseline correction was performed using the sample buffer at 25 °C. Fluorescence emission spectra were measured with a Fluoromax-4 fluorescence spectrophotometer (Horiba). A long-pass filter allowing the transmission of light with wavelengths longer than 600 nm was placed in the emission window.

### Atomic coordinates

Atomic coordinates of the chlorophyll trimers in Pc-frLHC were obtained from the cryo-EM Pc-frLHC complex structure (PDB code, 8HW1) (15). Atomic partial charges of amino acids were adopted from the all-atom CHARMM22 27 parameter set (35). Atomic charges for chlorophyll *a* were adopted from previous studies on PSI (36). Atomic charges of violaxanthin and loroxanthin were determined by fitting the electrostatic potential in the vicinity of these molecules using the restrained electrostatic potential procedure (37) (Tables S4 and S5). Electronic densities were calculated after geometry optimization using the density functional theory with the B3LYP functional and 6-31g∗ basis sets, implemented in the JAGUAR program (38). For nonpolar CH_*n*_ groups in cofactors (*e.g.*, phytol chains of Chl*a*), a charge of +0.09 was assigned to nonpolar H atoms as typically defined in CHARMM.

### Protonation pattern

The protonation states of titratable residues were calculated by solving the linear Poisson-Boltzmann equation using the MEAD program (39). To maintain consistency with previous computational results (*e.g.*, (36, 40, 40)), all calculations were performed at 300 K, pH 7.0, and an ionic strength of 100 mM. The dielectric constants used were four for the protein interior and 80 for water. The p*K*_a_ values of titratable sites in the protein were determined by adding the calculated p*K*_a_ shifts relative to a reference system to known reference p*K*_a_ values: 12.0 for Arg, 4.0 for Asp, 9.5 for Cys, 4.4 for Glu, 10.4 for Lys, 9.6 for Tyr (41), and 7.0 and 6.6 for the N_ε_ and N_δ_ atoms of His, respectively (42, 43, 44). All other titratable sites were equilibrated to the protonation state of the target site during titration, performed using Monte Carlo sampling *via* Karlsberg (45). The linear Poisson-Boltzmann equation was solved using a three-step grid-focusing procedure with resolutions of 2.5 Å, 1.0 Å, and 0.3 Å. Monte Carlo sampling yielded the probabilities ([protonated] and [deprotonated]) for each site. The resulting protonation patterns were subsequently used for QM/MM calculations.

### QM/MM calculations

To calculate the electronic excitation states of chlorophylls, QM/MM-optimized geometries are required. Geometry optimization was performed using the electrostatic embedding QM/MM scheme, explicitly considering the electrostatic and steric effects of the protein environment. The Qsite (46) program was used for QM/MM calculations, employing the restricted density functional theory method with the B3LYP functional and LACVP∗ basis sets (LANL2DZ [double ζ quality basis set with Los Alamos effective core potential] for Mg atoms and 6-31G∗ for other atoms) (47). The QM region was defined as the chlorophyll molecule excluding the phytyl tail while including the axial ligand group if present (Table S2). All atomic coordinates in the QM region were fully relaxed. In the MM region, H atom positions were optimized using the OPLS2005 force field (48), while the heavy atom positions were held fixed.

Electronically excited states of chlorophylls were calculated based on the optimized geometries using the time-dependent density functional theory (TDDFT) method with 6-31G∗ basis sets and the CAM-B3LYP functional (49). The range-separation parameters were set to *μ* = 0.14 (50), *α* = 0.19, and *β* = 0.46. For calculations in the protein environment, a TDDFT-QM/MM/polarizable continuum model (PCM) approach (2, 8, 51) was used. The QM region was defined as chlorophyll monomer, dimer, or trimer excluding the phytyl tails and including any axial ligand groups present (Table S2). This approach explicitly considered the electrostatic interactions of the protein environment in the presence of bulk water, modeled with a dielectric constant of 78 and a solvent radius of 3.0 Å. The CHARMM force field (35) was used for the MM atoms. The QuanPol method (52), implemented in the GAMESS program (53), was used to describe interactions between QM and MM atoms with Lennard–Jones parameters.

The calculated absorption energy for chlorophyll (*E*_calc_) was adjusted to reproduce the experimentally measured absorption (*E*_abs_), using an empirical equation correction derived for TDDFT-QM/MM calculations, applicable to arbitrary solvents (50):

(1) $$E_{\mathrm{abs}}\;\left(\text{meV}\right)=E_{\mathrm{calc}}\;–\;253$$

## Data availability

All of the data supporting the findings of this study are available within the paper and the supporting information.

## Supporting information

This article contains supporting information (13, 15, 33).

## Conflict of interest

The authors declare that they have no conflicts of interest with the contents of this article.
