# Supplementary material for: Identification and design principles of far-red–absorbing chlorophyll in the light-harvesting complex
Source: J Biol Chem. 2025 Apr 18;301(6):108518. doi: 10.1016/j.jbc.2025.108518 (PMC12148475; doi:10.1016/j.jbc.2025.108518)
Supplement: Supporting information 1 [file mmc1.pdf]

Supporting Information

Identification and design principles of far-red-absorbing chlorophyll in the light-harvesting complex

Keisuke Saito*[1,2], Makiko Kosugi[3,4], Linhao Qiu[1], Jun Minagawa[3,4], and Hiroshi Ishikita*[1,2]

1) Department of Applied Chemistry, The University of Tokyo, 7-3-1 Hongo, Bunkyo-ku, 113-8654 Tokyo, Japan

2) Research Center for Advanced Science and Technology, The University of Tokyo, 4-6-1 Komaba, Meguro-ku, 153-8904 Tokyo, Japan

3) Division of Environmental Photobiology, National Institute for Basic Biology, 38 Nishigonaka, Myodaiji, Okazaki, 444-8585 Aichi, Japan

4) Graduate Institute for Advanced Studies, SOKENDAI, 38 Nishigonaka, Myodaiji, Okazaki 444-8585, Aichi, Japan

* CORRESPONDING AUTHOR:

K. Saito **E-mail:** ksaito@appchem.t.u-tokyo.ac.jp

H. Ishikita **E-mail:** hiro@appchem.t.u-tokyo.ac.jp

Contents

11 pages

3 figures

6 tables

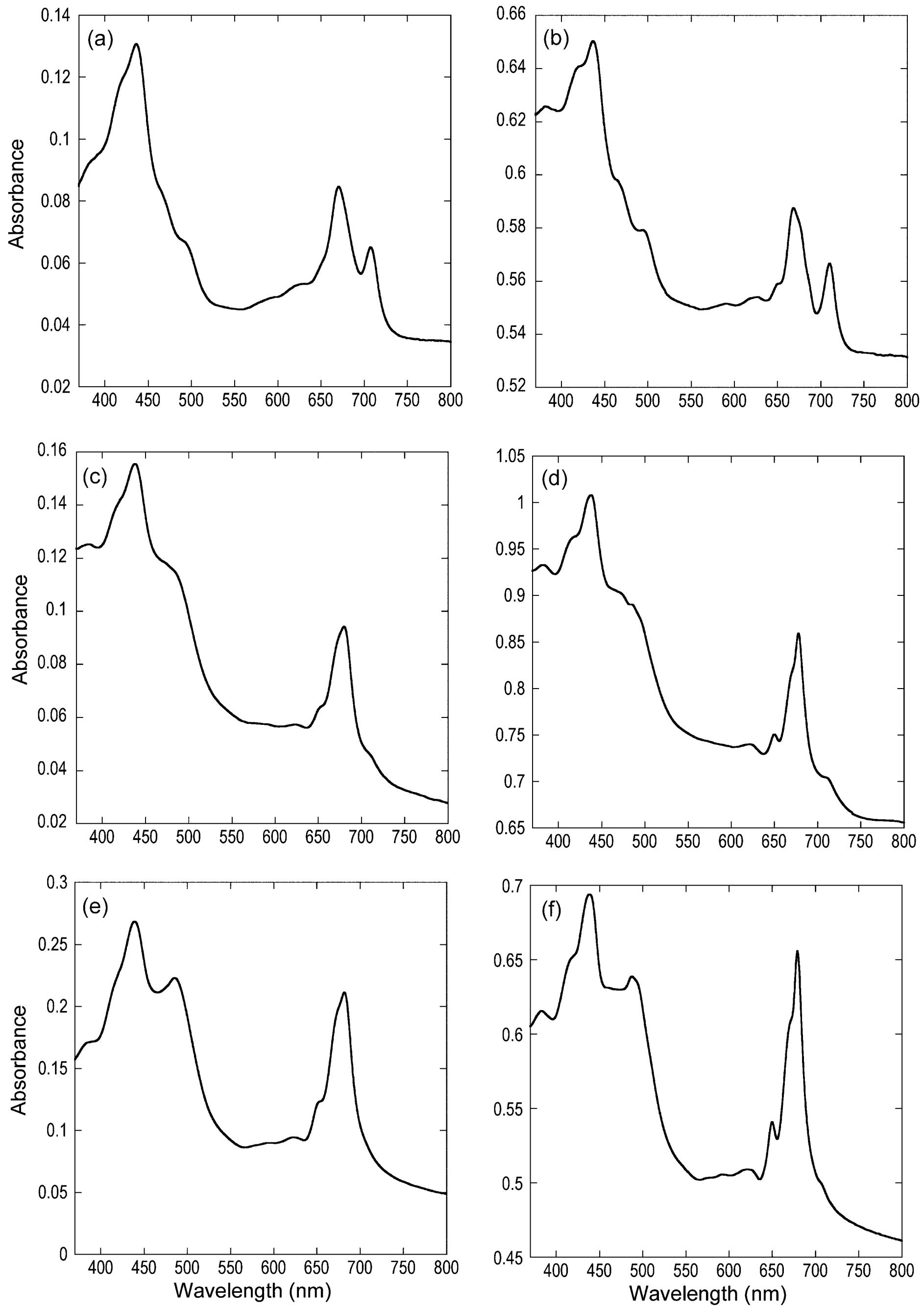

Figure S1. Absorbance spectra. (a) Purified Pc-frLHC at 298 K. (b) Purified Pc-frLHC at 93 K. (c) Isolated thylakoid membranes from *P. crispa* at 298 K. (d) Isolated thylakoid membranes from *P. crispa* at 93 K. (e) Isolated thylakoid membranes from *Coccomyxa* sp. Obi at 298 K. (f) Isolated thylakoid membranes from *Coccomyxa* sp. Obi at 93 K. The spectra shown are representative of multiple independent measurements that gave consistent results.

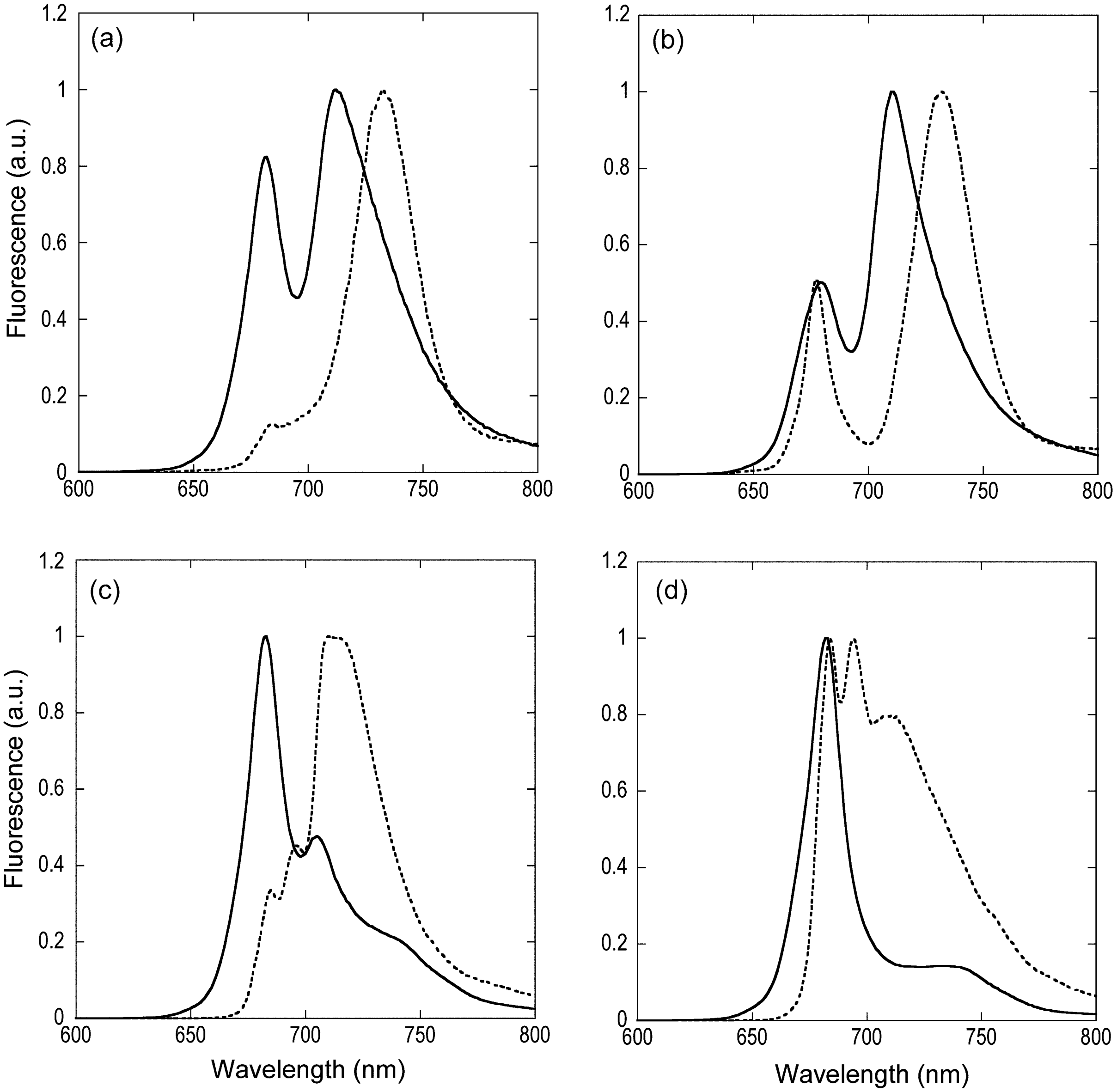

Figure S2. Fluorescence emission spectra measured at 298 K (solid lines) and 77K (dotted lines). (a) Thylakoid membranes isolated from *P. crispa*. (b) Purified Pc-frLHC. (c) Thylakoid membranes isolated from *Coccomyxa* sp. Obi containing long-wavelength chlorophylls. (d) Thylakoid membranes isolated from *Coccomyxa* with a low amount of long-wavelength chlorophylls. Each spectrum was normalized at the highest fluorescence intensity (set as 1). The spectra shown are representative of multiple independent measurements that gave consistent results.

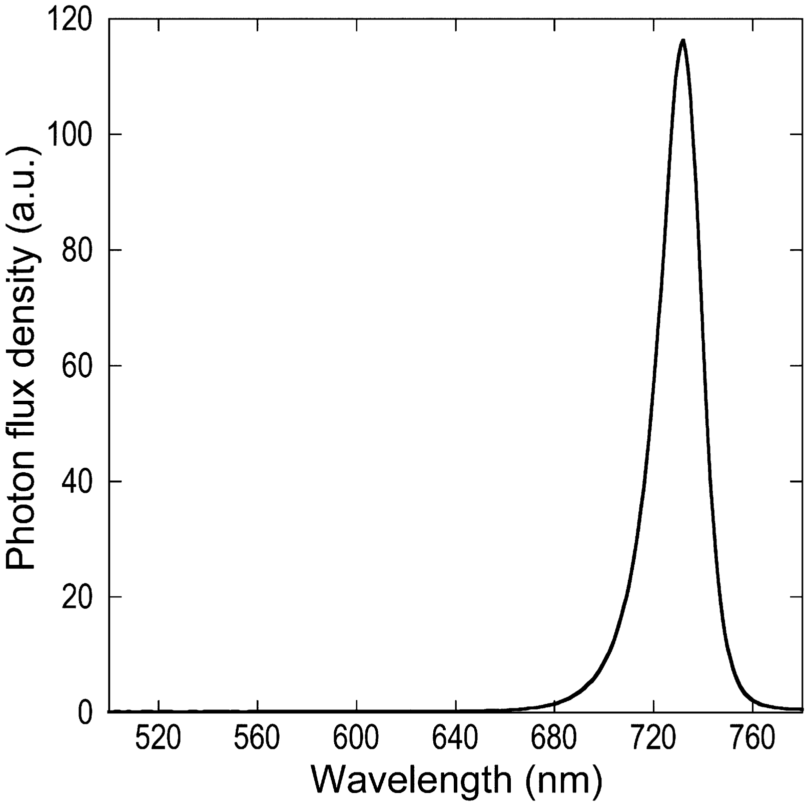

Figure S3. Spectrum of the far-red LED illumination used to induce red-shifted chlorophylls in *Coccomyxa* sp. Obi. The spectrum shows a peak wavelength at 732 nm.

Table S1. Calculated absorption energy (E_{abs}) for monomeric, dimeric, and trimeric chlorophylls in the Pc-frLHC structure (meV). The shifts in E_{abs} from water (1822 eV) to the Pc-frLHC protein environment (ΔE_{abs}) are decomposed into contributions from: (i) atomic charges of the axial ligand group, (ii) atomic charges of the other protein environment, (iii) loss of solvation at the binding site in the protein environment, and (iv) distortion of chlorin ring caused by the protein environment. ΔE_{abs} is exactly the sum of these four components. –: not determined. See Table 2 for calculated absorption wavelengths in nm.

		E_{abs}		ΔE_{abs}				
**chlorophyll**	**ligand**		**(water)**	**shift (total)**	**-charge (ligand)** [a]	**-charge (others)** [b]	**-solvation loss**[c]	**-ring distortion**[d]
601	–	1862	(1822)	40	6	2	55	-23
602	Glu124	1807	(1822)	-15	-26	15	34	-38
603	Asn127	1857	(1822)	35	0	10	32	-7
604	–	1849	(1822)	27	-10	5	53	-21
609	Glu178	1826	(1822)	4	-1	-9	42	-28
610	Glu219	1867	(1822)	45	0	-9	53	1
611	Glu63	1798	(1822)	-24	-10	7	14	-35
612	Asn222	1820	(1822)	-2	0[e]	-12	28	-18
613	Gln236	1867	(1822)	45	-14	-5	43	21
614	His251	1853	(1822)	31	0[f]	-6	25	12
708	His171	1803	(1822)	-19	-26	2	40	-35
603-609	–	1756	(1822)	-66	–	–	–	–
609-708	–	1773	(1822)	-49	–	–	–	–
611-612	–	1809	(1822)	-13	–	–	–	–
613-614	–	1793	(1822)	-29	–	–	–	–
708-614’	–	1795	(1822)	-27	–	–	–	–
			(1822)					
603-609-708	–	1745	(1822)	-77	–	–	–	–

[a] C – D in E_{abs} of Table S6.

[b] A – B in E_{abs} of Table S6.

[c] B – C in E_{abs} of Table S6.

[d] D – E in E_{abs} of Table S6.

[e]No axial ligand exists in the cryoEM structure(1).

Table S2. Comparisons of chlorophylls and ligand groups in Pc-frLHC (PDB: 8HW1), CrLhca2 (PDB: 7DZ7), and spinach LHCII (PDB: 1RWT). For Pc-frLHC, the numbering of chlorophylls provided in the PDB is listed in the third column (PDB #) for clarity, as the numbering used in ref. (1) is inconsistent. For CrLhca2 and spinach LHCII, the numbering in the PDB is consistent with refs (2,3). Corresponding chlorophylls among the three proteins are listed in the same line. PG: phosphatidylglycerol.

**Pc-frLHC**			**CrLhca2**		**Spinach LHCII**	
	**ligand**	**(PDB #)**		**ligand**		**ligand**
**Stroma**						
Chl*a* 601	–	(601)	Chl*a* 601	Trp31 (C=O)	Chl*b* 601	Tyr24 (C=O)
					Chl*b* 608	H_2O
Chl*a* 609	Glu178	(605)	Chl*a* 609	Glu222	Chl*b* 609	Glu139
Chl*a* 603	Asn127	(603)	Chl*a* 603	Asn73	Chl*a* 603	His68
Chl*a* 602	Glu124	(602)	Chl*a* 602	Glu70	Chl*a* 602	Glu65
Chl*a* 610	Glu219	(606)	Chl*a* 610	Glu157	Chl*a* 610	Glu180
Chl*a* 611	Glu63	(607)	Chl*a* 611	PG	Chl*a* 611	PG
Chl*a* 612	Asn222	(608)	Chl*a* 612	Asn160	Chl*a* 612	Asn183
**Lumen**						
					Chl*b* 605	Val119 (C=O)
			Chl*a* 606	(Glu114) [a]	Chl*b* 606	H_2O
			Chl*a* 607	–	Chl*b* 607	H_2O
Chl*a* 604	–	(604)	Chl*a* 604	–	Chl*a* 604	H2O
Chl*a* 613	Gln236	(609)	Chl*a* 613	Gln174	Chl*a* 613	Gln197
Chl*a* 614	His251	(610)	Chl*a* 614	His189	Chl*a* 614	His212
Chl*a* 708	His171	(611)				
			Chl*a* 616	Glu206		

[a] The O...Mg distance is 4.6 Å, which is too long to consider Glu114 as the axial ligand.

Table S3. Calculated induced dipole moments upon excitation of chlorophylls in Pc-frLHC (Debye). $\Delta\mu_{Mg\text{-}Mg}$ represents the projection of the excitation-induced dipole moment $\boldsymbol{\Delta\mu}$ along the Mg-Mg axis of the dimer (Figure 3d), where $\boldsymbol{\Delta\mu} = \boldsymbol{\mu_e} - \boldsymbol{\mu_g}$; $\boldsymbol{\mu_e}$ and $\boldsymbol{\mu_g}$ are permanent dipole moments of the excited and ground states, respectively.

	**Ligand**	$\boldsymbol{\Delta\mu_{Mg\text{-}Mg}}$
Chl*a* 603-609-708	Glu-Asn-His	2.03[a]
Chl*a* 603-609	Glu-Asn	3.05
Chl*a* 613-614	Gln-His	0.99
Chl*a* 611-612	Asn-Glu	0.88
Chl*a* 708-611’	His-Asn	0.47
Chl*a* 708-614	His-His	0.15

[a] Projection of $\Delta\mu$ along the Mg-Mg axis for the Chl*a* 603-609 dimer.

Table S4. Atomic partial charges of violaxanthin.

C1	0.262	C17	-0.308	C29	0.102
C2	-0.148	H17A	0.079	C30	-0.248
H2A	0.054	H17B	0.079	H30	0.127
H2B	0.054	H17C	0.079	C31	0.001
C3	0.292	C18	-0.457	H31	0.118
H3	0.008	H18A	0.122	C32	-0.308
O3	-0.612	H18B	0.122	H32	0.151
HO3	0.381	H18C	0.122	C33	0.215
C4	-0.239	C19	-0.173	C34	-0.254
H4A	0.068	H19A	0.057	H34	0.117
H4B	0.068	H19B	0.057	C35	-0.097
C5	0.349	H19C	0.057	H35	0.141
O4	-0.338	C20	-0.248	C36	-0.308
C6	0.026	H20A	0.076	H36A	0.079
C7	-0.114	H20B	0.076	H36B	0.079
H7	0.098	H20C	0.076	H36C	0.079
C8	-0.129	C21	0.262	C37	-0.308
H8	0.110	C22	-0.148	H37A	0.079
C9	0.102	H22A	0.054	H37B	0.079
C10	-0.248	H22B	0.054	H37C	0.079
H10	0.127	C23	0.292	C38	-0.457
C11	0.001	H23	0.008	H38A	0.122
H11	0.118	O23	-0.612	H38B	0.122
C12	-0.308	HO23	0.381	H38C	0.122
H12	0.151	C24	-0.239	C39	-0.173
C13	0.215	H24A	0.068	H39A	0.057
C14	-0.254	H24B	0.068	H39B	0.057
H14	0.117	C25	0.349	H39C	0.057
C15	-0.097	O24	-0.338	C40	-0.248
H15	0.141	C26	0.026	H40A	0.076
C16	-0.308	C27	-0.114	H40B	0.076
H16A	0.079	H27	0.098	H40C	0.076
H16B	0.079	C28	-0.129		
H16C	0.079	H28	0.110	**total**	**0.000**

Table S5. Atomic partial charges of loroxanthin.

C1	0.085	C13	-0.075	H29	0.016
C2	-0.293	H13	0.142	C30	0.151
C3	0.492	C14	-0.264	C31	-0.369
C4	-0.402	H14	0.140	H31	0.160
H41	0.097	C15	0.208	C32	0.365
H42	0.097	C16	-0.262	H32	0.000
C5	0.317	H16	0.121	C33	-0.277
H5	0.000	C17	-0.100	H331	0.033
C6	-0.139	H17	0.145	H332	0.033
H61	0.084	C18	-0.097	C34	0.570
H62	0.084	H18	0.144	C35	-0.516
C7	-0.329	C19	-0.262	H351	0.116
H71	0.072	H19	0.119	H352	0.116
H72	0.072	C20	0.216	H353	0.116
H73	0.072	C21	-0.255	C36	-0.516
C8	-0.329	H211	0.078	H361	0.116
H81	0.072	H212	0.078	H362	0.116
H82	0.072	H213	0.078	H363	0.116
H83	0.072	C22	-0.298	C37	-0.381
C9	0.069	H22	0.147	H371	0.108
H9	0.070	C23	0.003	H372	0.108
O1	-0.602	H23	0.114	H373	0.108
HO1	0.376	C24	-0.318	C38	-0.248
C	-0.188	H24	0.144	H381	0.075
1HXT	0.052	C25	0.268	H382	0.075
2HXT	0.052	C26	-0.291	H383	0.075
3HXT	0.052	H261	0.083	C39	0.232
O	-0.642	H262	0.083	H391	0.000
HO	0.395	H263	0.083	H392	0.000
C10	-0.207	C27	-0.337	O2	-0.593
H10	0.118	H27	0.148	HO2	0.382
C11	0.039	C28	-0.107		
C12	-0.199	H28	0.115		
H12	0.130	C29	0.011	**total**	**0.000**

Table S6. Calculated absorption energies E_{abs} of chlorophyll monomers (meV)

chlorophyll	E_{abs} (A)	E_{abs} (B)	E_{abs} (C)	E_{abs} (D)	E_{abs} (D)
environment	protein	uncharged protein	water	water	water
ligand	present	present	present	absent	absent
distortion	present[a]	present[a]	present[a]	present[a]	absent[b]
603	1862	1860	1805	1799	(1822)
609	1807	1792	1758	1784	(1822)
708	1857	1847	1815	1815	(1822)
611	1849	1844	1791	1801	(1822)
612	1826	1835	1793	1794	(1822)
613	1867	1876	1823	1823	(1822)
614	1798	1791	1777	1787	(1822)
601	1820	1832	1804	1804	(1822)
602	1867	1872	1829	1843	(1822)
604	1853	1859	1834	1834	(1822)
610	1803	1801	1761	1787	(1822)

[a] Optimized geometry in the protein environment.

[b] Optimized geometry in vacuum.

References

1. Kosugi, M., Kawasaki, M., Shibata, Y., Hara, K., Takaichi, S., Moriya, T., Adachi, N., Kamei, Y., Kashino, Y., Kudoh, S., Koike, H., and Senda, T. (2023) Uphill energy transfer mechanism for photosynthesis in an Antarctic alga. *Nat Commun* **14**, 730
2. Pan, X., Tokutsu, R., Li, A., Takizawa, K., Song, C., Murata, K., Yamasaki, T., Liu, Z., Minagawa, J., and Li, M. (2021) Structural basis of LhcbM5-mediated state transitions in green algae. *Nat Plants* **7**, 1119-1131
3. Liu, Z., Yan, H., Wang, K., Kuang, T., Zhang, J., Gui, L., An, X., and Chang, W. (2004) Crystal structure of spinach major light-harvesting complex at 2.72 Å resolution. *Nature* **428**, 287-292
